# Supplementary material for: Fer3 is uniquely expressed in NotchOFF hemilineages, where it promotes interneuron identity
Source: Development. 2025 Nov 14;152(22):dev205118. doi: 10.1242/dev.205118 (PMC12669965; doi:10.1242/dev.205118)
Supplement: Supplementary information [file develop-152-205118-s1.pdf]

Table S1. Key resources table

Reagent type (species) or resource	Designation	Source or reference	Identifiers	Additional information
Genetic Reagent (*D. melanogaster*)	en-Gal4	BDSC	RRID:BDSC_99568	Fig. 1
Genetic Reagent (*Drosophila melanogaster*)	vnd-VP16; R16H05-DBD	(Pollington and Doe, 2025)		Short name: NB5-2-Gal4<br>Figs 1-4
Genetic Reagent (*D. melanogaster*)	Gal4-T2A-fd96Ca	(Anderson et al., 2025 preprint)		Short name: NB7-1-Gal4<br>Figs 1-3
Genetic Reagent (*D. melanogaster*)	elav-Gal4	BDSC	RRID:BDSC_8765	Fig. 3
Genetic Reagent (*D. melanogaster*)	lbe-Gal4	(Baumgardt et al., 2009)		Short name: NB5-6-Gal4<br>Fig. 4
Genetic Reagent (*D. melanogaster*)	*ac-VP16,gsb-DBD R25A05-KillerZipper*	(Seroka and Doe, 2019)		Short name: NB7-1-$Gal4^{KZ}$<br>Fig. 4
Genetic Reagent (*D. melanogaster*)	10XUAS-myr::smGdP-HA	BDSC	RRID:BDSC_62145	Figs 1-4
Genetic Reagent (*D. melanogaster*)	UAS-RedStinger	BDSC	RRID:BDSC_8547	Fig. 4
Genetic Reagent (*D. melanogaster*)	UAS-N^{intra}	(Larkin et al., 1996; Truman et al., 2010)		Fig. 3
Genetic Reagent (*D. melanogaster*)	*$spdo^{C55}$*	BDSC	RRID:BDSC_4374	Short name: *spdo* mutant<br>Fig. 3
Genetic Reagent (*D. melanogaster*)	(FRT.stop) lexA.p65-T2A-Fer3	This work		Short name: Fer3 loss of function mutant<br>Fig. 4
Genetic Reagent (*D. melanogaster*)	UAS-Fer3	This work		Fig. 4
Genetic Reagent (*D. melanogaster*)	UAS-Fer3-p65	This work		Short name: Fer3-activator (Fer3:AD)<br>Fig. 4
Antibody, polyclonal	Rabbit anti-Ase	(Weng et al., 2010)		1:1000
Antibody, polyclonal	Rabbit anti-Cas	(Mellerick et al., 1992)		1:1000
Antibody, polyclonal	Guinea Pig anti-Dbx	Doe Lab		1:200
Antibody, monoclonal	Rat anti-elav	DHSB, Iowa City, IA	RRID:AB_528218	1:100
Antibody, polyclonal	Guinea Pig anti-Fer3	This work		10 µg/ml
Antibody, polyclonal	Chicken anti-GFP	Aves Labs, Davis, CA	RRID:AB_2307313	1:1000
Antibody, monoclonal	Mouse anti-HA	BioLegend, San Diego, CA	901513	1:100
Antibody, monoclonal	Rat anti-HA	MilliporeSigma, St. Louis, MO	11867423001	1:100
Antibody, monoclonal	Mouse anti-Hb	abcam, Eugene, OR	ab197787	1:200
Antibody, polyclonal	Rabbit anti-Hey	Doe Lab		5 µg/ml

Antibody	Guinea Pig anti-Kr	Doe Lab		1:500
Antibody, monoclonal	Rat anti-Pdm2	abcam, Eugene, OR	ab201325	1:100
Antibody, secondary	Alexa Fluor® 488 conjugated-AffiniPure Donkey IgG (H+L)	Jackson ImmunoResearch, West Grove, PA		1:200
Antibody, secondary	Alexa Fluor® 555 conjugated-AffiniPure Donkey IgG (H+L)	Jackson ImmunoResearch, West Grove, PA		1:200
Antibody, secondary	Alexa Fluor® 647 conjugated-AffiniPure Donkey IgG (H+L)	Jackson ImmunoResearch, West Grove, PA		1:200
HCR probe	Fer3 RNA probe	IDT		2x probe conc. used (see protocol below)
HCR amplifier	B2-647	Molecular Instruments		
